# Safe surgery: validation of pre and postoperative checklists^1^

**DOI:** 10.1590/1518-8345.1854.2907

**Published:** 2017-07-10

**Authors:** Francine Taporosky Alpendre, Elaine Drehmer de Almeida Cruz, Ana Maria Dyniewicz, Maria de Fátima Mantovani, Ana Elisa Bauer de Camargo e Silva, Gabriela de Souza dos Santos

**Affiliations:** 2 Doctoral student, Universidade Federal do Paraná, Curitiba, PR, Brazil. RN, Hospital de Clínicas, Universidade Federal do Paraná, Curitiba, PR, Brazil.; 3 PhD, Adjunct Professor, Universidade Federal do Paraná, Curitiba, PR, Brazil.; 4 PhD, Professor, Universidade Federal do Paraná, Curitiba, PR, Brazil.; 5 PhD, Associate Professor, Universidade Federal do Paraná, Curitiba, PR, Brazil.; 6 PhD, Adjunct Professor, Departamento de Enfermagem, Universidade Federal de Goiás, Goiânia, GO, Brazil.; 7 Master’s student, Universidade Federal do Paraná, Curitiba, PR, Brazil. RN, Instituto De Neurologia de Curitiba, Curitiba, PR, Brazil.

**Keywords:** Patient Safety, Checklist, Validation Studies

## Abstract

**Objective:**

to develop, evaluate and validate a surgical safety *checklist* for patients in the pre and postoperative periods in surgical hospitalization units.

**Method:**

methodological research carried out in a large public teaching hospital in the South of Brazil, with application of the principles of the Safe Surgery Saves Lives Programme of the World Health Organization. The checklist was applied to 16 nurses of 8 surgical units and submitted for validation by a group of eight experts using the Delphi method online.

**Results:**

the instrument was validated and it was achieved a mean score ≥1, level of agreement ≥75% and Cronbach’s alpha >0.90. The final version included 97 safety indicators organized into six categories: identification, preoperative, immediate postoperative, immediate postoperative, other surgical complications, and hospital discharge.

**Conclusion:**

the Surgical Safety Checklist in the Pre and Postoperative periods is another strategy to promote patient safety, as it allows the monitoring of predictive signs and symptoms of surgical complications and the early detection of adverse events.

## Introduction

Nurses’ decision-making processes encompass knowledge in the area of care and management, with an emphasis on patient care. However, for their success, they must occur in line with planning and evaluation, based on an appropriate information system. The information within a health service not only favors decision making, but also the structuring of innovative strategies that significantly help in the management. This is the challenge, from a wider perspective, for the insertion and performance of nurses in the organizational structure of health systems^1^. 

Among the management actions is the decision making of the nurses, it is possible to highlight those actions related to patient safety aiming at the prediction and reduction of complications, as well as the early detection of aggravations and adverse events in the postoperative period^2^. In this context, the development of tools that provide information, such as *checklists*, may promote the early identification of the most frequent problems in the planning of nursing care during hospital stay, discharge plan development and guidance on home care^3^. 

The initial milestone, which demonstrated the benefits of using a *checklist* for the safety of surgical patients, was a study conducted by experts of the World Health Organization (WHO) in eight countries (Canada, India, Jordan, Philippines, New Zealand, Tanzania, England and USA). In total 7,688 patients were investigated, of which 3,733 were investigated prior to the use of the *checklist* and 3,955 after filling out the *checklist*, which showed a 36% reduction in surgical complications, 47% in mortality, 50% in infection rates and 25% in the need for a new surgical intervention. It was concluded that the use of the *checklist* practically doubled the possibility of using safe care standards during surgical treatment of the patients^4^.

These results on the use of the Surgical Safety *Checklist* (SSC) were highlighted in the WHO Second Global Patient Security Challenge. In Brazil, the Ministry of Health has implemented the Safe Surgeries Programme and recommends the use of SSC before anesthetic induction, before the surgical incision and at the end of the surgery, before the patient leaves the operating room^5^.

A systematic review concluded that surgical safety *checklists* are considered instruments to coordinate care, promote team union and reduce postoperative complications. Such complications frequently involve pneumonia, pulmonary embolism, deep vein thrombosis, surgical site infection, unplanned return to the operating room, blood loss, death, suture dehiscence, cerebrovascular accident, acute myocardial infarction, vascular graft failure, systemic inflammatory response syndrome, septic shock, cardiac arrest and acute renal failure^6^.

Other studies show that the use of *checklists* is a practice encouraged by reducing memory and intuition dependence^7^ and reducing errors^8^, thus becoming synonymous with best practice in high-risk areas^9^. These verification tools can revolutionize the way knowledge is put into practice, as well as being a basic and cost-effective resource for health services^10^.

Considering that the WHO SSC model is applicable in surgical centers, that is, in perioperative moments (before, during and after surgery), it is justified the need for a specific *checklist* in the pre and postoperative periods in the hospital surgical units. This allows identifying the appropriate preparation of the patients before their referral to the surgical center, as well as the predictive signs of postoperative complications. 

Another study concluded that the prevention of problems related to the safety of surgical patient should also be focused on the pre and postoperative periods, as it is estimated that 19% of incidents are associated with the organization of services and care^11^.

The WHO recommends the development of new *checklists* for other in-hospital services, as a way of stimulating the safety culture^5^. Thus, based on the international recommendations for safe surgeries, it is the ethical responsibility of the nursing professional to fill the gap identified in relation to the verification of safety elements before the referral of the patient to the surgical center, as well as the identification of predictive factores for postoperative complications.

The objective of this study was to develop, evaluate and validate a surgical safety *checklist* for patients in the pre and postoperative periods in surgical hospitalization units.

## Methods

Methodological study, with a quantitative approach, conducted in a large public teaching hospital, located in the South Region of Brazil, from March 2013 to October 2014, with the participation of 16 nurses of eight surgical services (Orthopedics and Traumatology, General Surgery, Digestive System Surgery, Urology, Plastic Surgery, Liver Transplantation, Pediatric Surgery and Neurosurgery). 

The development, assessment and validation of the *checklist* for patients in the pre and postoperative periods (SSCPP) was guided by the principles of the Safe Surgery Saves Lives Programme of the WHO: simplicity, applicability and measurement capacity of the *checklist*-type instrument to the safe surgery^5^. Its implementation followed the quality management proposals, in line with the model used in the hospital focus of this research, according to the phases of the PDCA Cycle (*Plan, Do, Check, Act*)^12^. 

The methodological steps of the implementation of the PDCA Cycle are presented as follows. 

### (1) P (Plan) - Planning Phase

The Planning Phase consisted of three meetings: the first one with nurses of surgical units, for awareness on the surgical safety, identification of gaps and analysis of problems related to the surgical safety of patients in hospitalization units, presentation, agreement with the research project and signing of the Informed Consent Form (TCLE). The inclusion criteria were: nurses who have been working for more than four weeks in a surgical unit and with a 20 hours weekly shift or over; as exclusion criteria: nurses on probationary period, on vacation, or nurses away from work on sick leave. The sample consisted of 16 nurses, all professionals of 8 surgical units. Two other meetings took place in continuity with the Action Plan, for the preparation and implementation of a pilot test of the *checklist*. 

### (2) D ( Do) - Development Phase

Two actions were taken in this phase: a) together with the participating nurses, the researchers identified and listed the items for version 1 of the *checklist*; b) two workshops were held with the nurses to improve version 1, resulting in version 2 of the *checklist*. 

### (3) C ( Check ) - Checking Phase

At this stage of the PDCA Cycle, version 2 of the *checklist* was subjected to a pilot test in the surgical units. The size of the sample was not set, and each nurse was asked to fill out as many instruments as possible during the pilot test period. The researchers have followed up the instrument by means of daily visits in the eight units; the researchers were responsible for clarifying doubts, encouraging the filling out the instrument and taking note of the suggestions in field diaries. 

At the end of the three-month period, suggestions were considered, such as words/sentences exchange, exclusion or inclusion of items in the instrument, completion of the necessary changes in the *checklist*, and definition of version 3. 

### (4) A (Act) - Action Phase

This phase refers to the submission of version 3 of the *checklist* to the validation process by the Committee of Experts, using the Delphi method, through an online panel to reach a consensus. It was established a minimum concordance of 70% for the results of the Average *Ranking* (AR) in the assessment^13^. This value was calculated by the sum of the frequencies of the responses, multiplied by the score assigned to each Likert scale response (weighting factor) and divided by the sum of the frequencies of each response using the weighted average of the frequencies. 

The data collection instrument was named the Experts Form and was composed of 23 questions, distributed in three blocks according to the Likert scale, with the following weights: (-2) Strongly Disagree, (-1) Disagree, (0) Indifferent, (+1) Agree and (+2) Strongly Agree. In the first block, with nine questions, the assessment focused on the effectiveness and comprehension of the writing of the items, application to the practice and contribution to the construction of knowledge. In the second block, with eight questions, the content of the questions related to patient safety, the need for inclusion and/or exclusion of items, the contributions of the instrument to care planning and the possibility of its replication were assessed. In the third block, with six questions, the assessment focused on the content, form, applicability and credibility of the *checklist*. On the side of the 23 questions, there was a specific space to write the comments of the experts.

Version 3 of the *checklist*, as well as the Experts Form, the invitation letter and the TCLE were sent by electronic mail, and a 14 days deadline was set out for feedback. The recruitment of the experts was carried out using the CNPq Lattes Platform, among those PhDs with *expertise* in surgical clinic, publications related to the safety of the surgical patient and who agreed to participate in the research*.*

Acceptance or rejection of the suggestions was based on their consistency with the WHO Safe Surgery Saves Lives Manual. The number of assessment rounds was not previously set, but there would be as many as necessary to reach consensus. 

To evaluate the reliability of the results, the Cronbach’s alpha test was used to correlate the answers of the experts when the options are staggered (-2, -1.0, +1, +2), as described in the Experts Form. In this respect, the following criteria was used: >0.90 - excellent; 0.81 to 0.90 - good; 0.71 to 0.80 - acceptable; 0.61 to 0.70 - questionable; 0.51 to 0.60 - poor and 0.41 to 0.50 - unacceptable. 

The development of the study followed the national and international standards of research ethics on human beings and was approved by the Ethics Committee under protocol number 546.183. The confidentiality of nurses and experts was ensured by the absence of identification throughout the data collection process.

## Results

The 16 nurses participating in the research, all women, with an average age of 40 years, postgraduated and more than 10 years of employment relationship with the hospital under study, worked in care and/or management positions in the surgical units.

The results of the methodological research are presented according to the progression and application of the PDCA Cycle and its respective phases.

(1) P *(Plan) -* Planning Phase - there were three meetings with the nurses participating in the study, from March to April 2013, when the Action Plans were written and approved aiming at the development and subsequent implementation of the pilot test of the *checklist*.

(2) D (*Do) -* Development Phase - in meetings with nurses, the main elements of care provided to patients in the pre and postoperative periods in clinical practice were listed. The relationships of care provided by the nurses resulted in the preliminary design of version 1 of the *checklist*, followed by workshops to improve that version, resulting in version 2 of the instrument. This phase took place from June 2013 and March 2014.

(3) C (*Check)* - Checking Phase - version 2 of the *checklist* was subjected to assessment and changes in the form and content, by means of a pilot test, with the application and filling out of 450 *checklists*, in eight surgical hospitalization services from April to May 2014. After analysis of the results of the instrument, the necessary changes suggested by the participating nurses were made, resulting in version 3, called the Surgical Safety *Checklist* in the Pre and Postperative periods (SSCPP).

(4) A *(Act) -* Action Phase - after the assessment and development phases of the SSCPP, the selection and recruitment of the experts for the validation of its form and content was initiated by using the Delphi method online. As for the training process of the group of Brazilian experts, 16 professionals were contacted, from the invitation letter, of which eight accepted to be part of this study. 

The committee of experts was composed of two professors of surgical nursing care, two specialists in surgical nursing, two nurses with specialization in patient safety and two surgeons. 

The SSCPP underwent two rounds of assessment by the experts, from June 2014, a consensus emerged and version 4 of the instrument is shown next. The results below refer to the responses of the Experts Form, with levels of agreement and *average ranking* of the three blocks of questions. 

Table 1 shows the assessment of the characteristics and purposes of the SSCPP, with level of agreement >75% and *average ranking* ≥1. 

**Table 1:** Average Ranking of the level of agreement in relation to the assessment of the characteristics and purposes of the SSCPP by the committee of experts (n=8). Curitiba, PR, Brazil, 2014

**Question**	**Agree %**	**Indifferent %**	**Disagree %**	***Average Ranking*** **Likert**
**Title helps readers to identify the information they will observe**	**100**	**0**	**0**	**1.38**
**Title is concise and attractive**	**88**	**12**	**0**	**1.25**
**Title corresponds to the Programme Safe Surgery Saves Lives**	**88**	**12**	**0**	**1.25**
**Practical application of the instrument**	**100**	**0**	**0**	**1.63**
**Knowledge of the researcher**	**100**	**0**	**0**	**1.88**
**It contributes to the knowledge construction**	**88**	**12**	**0**	**1.63**
**There is consistency or relation between the categories**	**88**	**12**	**0**	**1.25**
**There are superfluous details or elements that divert the attention of the reader**	**25**	**0**	**75**	**1.00**
**Text with appropriate size and positioning**	**76**	**12**	**12**	**1.00**

Table 2 shows the data of the assessment on the use of the SSCPP. The questions “Are there any items that need to be more detailed?”; “Is there any topic that should be included for completeness?” and “Is there any topic that should be excluded?” did not reach a minimum level of agreement of 70% and *average ranking* ≥1, in the first round of assessment by using the Delphi method. 

**Table 2:** Average ranking of the level of agreement of the possibility of using the SSCPP, by the committee of experts (n=8). Curitiba, PR, Brazil, 2014

**Question**	**Agree (%)**	**Indifferent (%)**	**Disagree (%)**	***Average Ranking*** **Likert**
*Checklist* **contributes to safety**	**100**	**0**	**0**	**1.63**
**There are elements** **that need further information**	**12**	**0**	**88**	**1.50**
**There are topics that should be included for completeness**	**12**	**0**	**88**	**1.38**
**There are topics that should be excluded**	**0**	**0**	**100**	**1.88**
*Checklist* **uses theoretical framework**	**100**	**0**	**0**	**1.50**
*Checklist* **is effective for planning and managing**	**100**	**0**	**0**	**1.63**
*Checklist* **will help prevent errors**	**88**	**12**	**0**	**1.50**
*Checklis* **t can be replicated**	**100**	**0**	**0**	**1.63**

After the first round of the Delphi method, at the suggestion of the experts, the expression “demarcated surgical site” was included in category II (prior to referral of the patient to the surgical center). In category III (return of the patient from the surgical center to the hospitalization unit), the experts requested space to describe the type and location of the drainage and inclusion of the word “others”, with space to write in the item related to permeable venous access. In category V (complications), the title was “Other postoperative complications”, and the types of shock were added - “septic”, “hypovolemic”, “cardiogenic”, “neurogenic” and “other” - with space to write. As for the exclusion, there were only changes in category V. The item PTE (Pulmonary Thromboembolism) was removed because the term VTE (Venous Thromboembolism) was already in the *checklist*; the item “Fall” was excluded because it was an incident and not a complication; and the item “dehiscence” was removed because it was already placed in category IV (immediate postoperative period), referring to the evaluation of the surgical site.

In general, the requests of the experts were more related to the presentation of the items than to the content of the instrument. It is inferred that the structure of the items of the manuscript corresponds to the need of checking the surgical safety. After modifications, the instrument was submitted to the second round of assessment by the Delphi method, and all questions assessed by the experts reached a level of agreement ≥88% and *average ranking* ≥1.38.

Table 3 shows the overall assessment of the SSCPP, with 100% approval in the attributes relevance, credibility and feasibility of implementation. The instrument was considered as appropriated for the work of the nurses in the pre and postoperative periods in the hospitalization units, a safe and reliable strategy, with easy and quick practical application.

**Table 3:** Average ranking of the level of agreement in the general assessment of the SSCPP, by the committee of experts (n=8). Curitiba, PR, Brazil, 2014

**Question**	**Agree (%)**	**Indifferent (%)**	**Disagree (%)**	***Average Ranking*** **Likert**
**Relevance**	**100**	**0**	**0**	**1.75**
**Credibility**	**100**	**0**	**0**	**1.75**
**Feasibility of implementation**	**100**	**0**	**0**	**1.75**
**Validity of the instrument**	**100**	**0**	**0**	**1.63**
**Logical organization of content**	**88**	**12**	**0**	**1.38**
**Professional interface and surgical patient**	**75**	**25**	**0**	**1.38**

The Cronbach’s alpha test was used to check the reliability of the SSCPP. The results showed an index of reliability of 0.9515 for the characteristics and purposes, 0.9396 for the possibilities of its use and 0.9858 for the general assessment.

**Figure 1:**
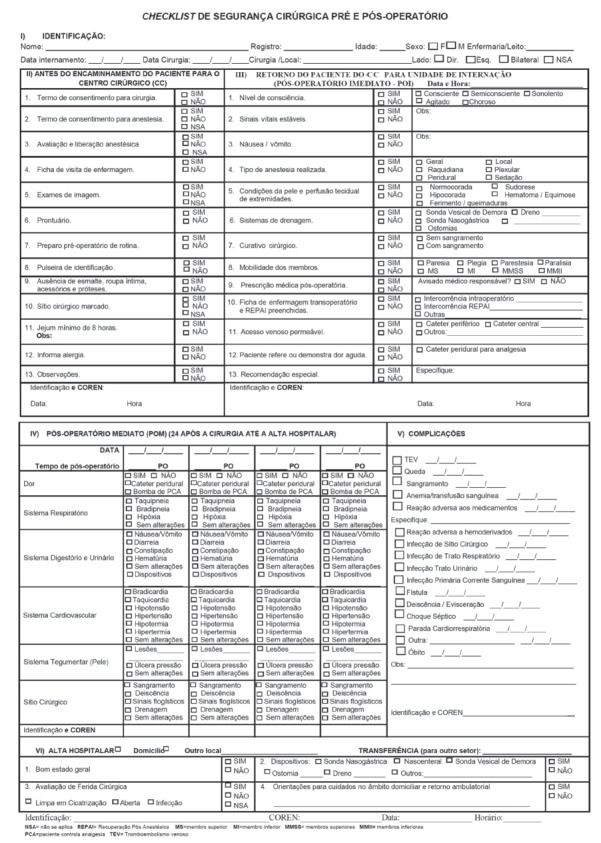
Surgical Safety Checklist of the Pre and Postperative periods (SSCPP). Curitiba, PR, Brazil, 2014

The experts validated the form and content of the SSCPP instrument, which includes 97 indicators of safety distributed in six categories: identification, preoperative, immediate postoperative, postoperative, other surgical complications and hospital discharge (Figure 1). 

The category Identification of the SSCPP includes information about the patient and other indicators of surgical safety, as suggested by WHO: right patient, right surgery and right side. These data provide minimal information, but aim to avoid adverse events and ensure the quality of care. 

In the category of the preoperative period, the actions of the nurse are listed to recognize and register items, such as: clinical history and other mandatory documents, imaging tests, preoperative preparation according to the surgical indication and identification devices.

In the category postoperative period, the SSCPP lists items such as: level of consciousness, stability of vital signs, nausea/vomiting, type of anesthesia, skin conditions and tissue perfusion of extremities, drainage systems, surgical dressing, mobility/sensitivity of the limbs, postoperative medical prescription, transoperative nursing record, postanesthetic recovery and recommendations. 

In the category of the immediate postoperative period, the SSCPP contemplates the evaluation of the patient’s pain: Respiratory, Digestive and Urinary, Cardiovascular and Tegumentary Systems, in addition to the evaluation of the surgical site.

The central focus of the category hospital discharge is the register and guidelines for home care, outpatient return, and post-surgical clinical reevaluation. It includes reports on general patient status, devices and surgical wound evaluation.

## Discussion

This study was an example of the feasibility of implementation of the PDCA Cycle as an organizational method, recommended for processes of continuous quality improvement. The PDCA Cycle is in line with the experimental scientific method, as it promotes the prediction of the results to be achieved, in addition to making it possible to measure the results and evaluate the impact of health interventions^12^.

The development of the phases of the PDCA Cycle (*Plan, Do, Check, Act*) to elaborate and evaluate a surgical safety *checklist* model, for the pre and postoperative periods, to be used in hospitalization units, was based on the participation and dialogue with nurses of surgical units. It served as a guide to bring to reality the needs and decisions of care, in a methodological and resolutive way. The joint efforts of researchers and nurses demonstrated their willingness and interest in innovate, bring practicality and give impact to the care actions of nursing teams.

For the nurse who participated in this study, this was a moment of convergence between the theoretical and managerial knowledge and the experience of professional practice, adding value to the research. The observation of attributes such as simplicity, applicability and the possibility of measurement contributed to the guidance on the development of the instrument, as well as to the possibility of turning a new working instrument more feasible. 

It should be considered that instruments such as PDCA help in improving safety quality, however, require from the nursing professionals the incorporation of behavioral changes, continuous expansion and dissemination of knowledge, development of skills and, consequently, changes in attitude. Although this instrument has been widely accepted in the area of health, providing structure for changes in the quality of services in the area in question, it is necessary to improve the patterns for the assessment aiming at their use, in a systematic and rigorous way^12^. 

It can be understood, then, that the use of PDCA served for the purposes of this study, the development and assessment of the SSCPP and its standardization for use, resulting in the version validated in the hospital. This is the conclusion of implementing the PDCA cycles, however, the implementation of this method and the assessment of the results of its impact should occur in practice.

In another study aiming at estimating the prevalence of risk in a surgical clinic, 750 hospitalizations were studied, among 5,672 records of incidents, and 218 were characterized as adverse events, as they caused harm to the patient. The most frequent incidents were acute postoperative pain, unplanned removal of tubular devices, probe and/or drain, failures in technical procedures requiring surgical intervention, as well as adverse and allergic reactions to medications, hospital infections; pressure ulcers, falls, inadequate maintenance of medical equipment, adverse reactions or lack of blood products and death^14^. In this context, the early identification of complications related to operative wound also contributes to guiding the care plan. Therefore, care planning and early identification of transoperative events support the development of the outcome indicators and monitoring of the quality of care and patient safety^5^.

A systematic review on the impacts and the implementation of a surgical *checklist* has demonstrated that the instrument can prevent perioperative errors and complications, reducing the rates of postoperative complications and mortality, besides providing a greater patient safety and improved communication among the care team^15^.

The results of the mentioned studies reveal that the use of *checklists* may contribute to reduce harms to patients. In addition to guiding the evaluation in the perioperative period, the information stored in these lists can also serve to feed databases, and provide support for health institutions and professionals^16^.

However, a validated instrument, as shown here, can provide more reliability for patient safety, reducing the costs of the health system and, in this scenario, the nurse is the professional who collaborates for this reality. In all areas of knowledge, including nursing, the development of validated assessment instruments is a complex process. However, it allows to recognize avoidable risk situations, to plan awareness actions, as well as to favor professional development. In addition, they call for reliability and consistency, as they reflect the quality of the measurement^17^.

The results of this study confirm the reliability of SSCPP and its contribution to the practice of surgical nursing. The confirmation of its reliability shows that the instrument serves to assess the quality of care, effectively manage care aiming at the identification of avoidable risks, and allows corrective actions and readjustment in the objectives through administrative and educational strategies^17^. 

The overall assessment of SSCPP was based on the information that, in North America, the implementation of this instrument caused an increase in the frequency of validation studies in the nursing area, increasing the relevance of assessment and measurement of the outcomes of this professional practice^18^. The Delphi method used in this research for the validation of the instrument, through consensus, was adequate and contributed to the form and content of the indicators, increasing the possibility of using this instrument in other health services.

It is important to highlight that the impacts of *checklists* are likely to be effective, depending on the implementation process of each hospital^19^. There might be several obstacles for achieving success in the implementation of a surgical *checklist*, such as organizational and cultural factors within each hospital. One strategy for achieving success is the continuous *feedback* from professionals of the service to the hospital administration in order to identify the factors that prevent the effective implementation of *checklists* for safe surgeries. In addition, the effectiveness of a *checklist* will depend on the ability of the institution’s leaders to implement it, and on the adaptation measures needed for each checking instrument^20^ ^-^ ^21^.

In this context, it is recommended including contents related to patient safety in the undergraduate and postgraduate nursing courses, as well as the training in health services^22^, since the *checklist* may serve as an example of good clinical practice and contribute to the development of safety behaviors.

This instrument may represent a guideline for pre and postoperative care in the hospitalization units, providing indicators to assess the quality of care and enabling the development of new strategies for the improment of health services. 

## Conclusion

The development of this study allowed the elaboration, assessment and validation of the SSCPP for surgical safety, based on the guidelines and objectives of the WHO Safe Surgery Saves Lives Programme. By consensus among the participants, it was considered that this tool is capable of assisting nurses in their clinical practice. 

At the end of this research, SSCPP was standardized for use in the institution. The SSCPP favours the adoption of preventive actions, as well as the monitoring of warning signs and symptoms, the early detection of complications and the minimization of risks for the patient. This instrument also contributes to the planning of the nursing interventions and improvement of the communication among the multiprofessional team on the care provided. The result of this research may be an effective and efficient instrument for the safety of the surgical patient, in addition to being adaptable to other health care contexts.

The implementation of this *checklist* only in a public and teaching hospital was a limitation of this study. It is recommended to use this instrument in other health services and, when necessary, adjust it according to the context of the institution.
